# Achievable information rate optimization in C-band optical fiber communication system

**DOI:** 10.1007/s12200-023-00072-5

**Published:** 2023-06-29

**Authors:** Zheng Liu, Tianhua Xu, Ji Qi, Joshua Uduagbomen, Jian Zhao, Tiegen Liu

**Affiliations:** 1 School of Precision Instrument and Opto-Electronics Engineering, Tianjin University, Tianjin, 300072 China; 2 School of Engineering, University of Warwick, Coventry, CV4 7AL UK; 3 Department of Electronic and Electrical Engineering, University College London, London, WC1E 7JE UK

**Keywords:** Optical fiber communication, Achievable information rate, Mutual information, Generalized mutual information

## Abstract

Optical fiber communication networks play an important role in the global telecommunication network. However, nonlinear effects in the optical fiber and transceiver noise greatly limit the performance of fiber communication systems. In this paper, the product of mutual information (MI) and communication bandwidth is used as the metric of the achievable information rate (AIR). The MI loss caused by the transceiver is also considered in this work, and the bit-wise MI, generalized mutual information (GMI), is used to calculate the AIR. This loss is more significant in the use of higher-order modulation formats. The AIR analysis is carried out in the QPSK, 16QAM, 64QAM and 256QAM modulation formats for the communication systems with different communication bandwidths and transmission distances based on the enhanced Gaussian noise (EGN) model. The paper provides suggestions for the selection of the optimal modulation format in different transmission scenarios.

**Graphical Abstract:**

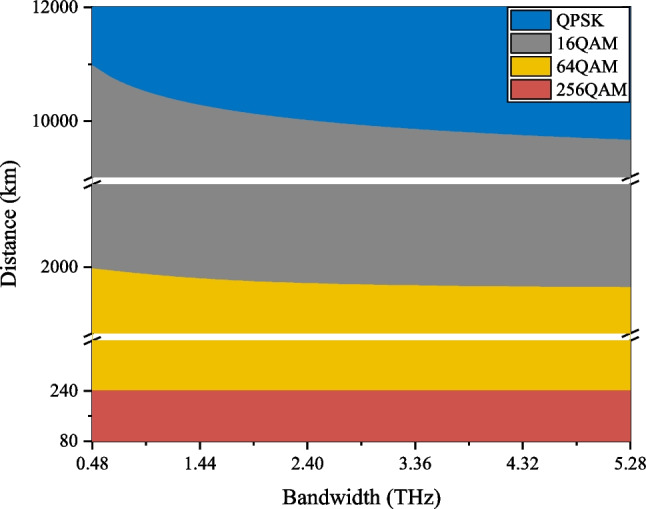

## Introduction

Over 95% of digital data traffic is carried over optical fiber networks [1]. The information transmission rate of optical fiber communication systems limits the communication rate of the global telecommunication networks. With the development of fiber communication technology, larger communication bandwidth and higher symbol rate are realized to transmit more bits within one second. However, severe nonlinear effects also occur and lead to fewer valid bits transmitted per second. Meanwhile, the equalization enhanced phase noise (EEPN) further decreases the signal quality [2]. In other words, the effective communication rate is limited by nonlinear effects and transmission noise. This phenomenon is more obvious when higher-order modulation formats are applied. Generally, a higher modulation format means a higher symbol error rate (SER) [3, 4]. However, using high-order modulation format can transmit more bits by each symbol. Therefore, it is not sufficient to use the signal-to-noise radio (SNR) to evaluate the performance of the communication system. To reasonably measure the communication capability, the transmission bit rate that the system can effectively support should be used as the metric. Generalized mutual information (GMI) can be used to measure the effective transmission bit rate of the system. For wavelength division multiplexed (WDM) systems, more channels can be used to transmit signals at the same time to achieve higher data rates. Although the larger bandwidth will further reduce the SNR due to the inter-channel interactions, the performance penalty is much less than the information rate gain arising from the use of more channels [5]. Therefore, this paper employs the number of bits effectively transmitted in one second as the metric of achievable information rate (AIR). The enhanced Gaussian noise (EGN) model is applied to analyze the performance of the optical fiber system under different conditions. Finally, the optimal modulation format is obtained by comprehensive analyses of different transmission scenarios. Discussions are conducted provide an optimization direction for future high-capacity optical fiber communication systems.

This paper evaluates different communication scenarios in terms of effective bit rates that can be efficiently transmitted. Such metric provides a fair comparison of systems, and the results have fundamental implications and provide insightful suggestions for follow-up research. The conclusions in this paper are based on systems without the application of forward error correction (FEC) techniques [6, 7]. Different types of FEC codes have different error-correction capabilities, and research of AIR in this case only needs to carry out a further-step based on our results. Moreover, the impact of introducing error correction codes on the transmission bit rate is linear, so the conclusions in this paper are insightful and applicable for systems with FECs.

This paper is arranged as follows. The GMI and MI are introduced in Sect. 2. Section 3 discusses the EGN model. The results and discussion can be found in Sect. 4 and some proposals for the future are presented in Sect. 5.

## Generalized mutual information

**Fig. 1:**
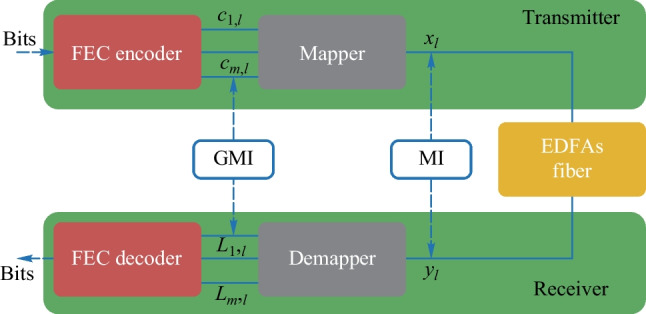
Schematic of MI and GMI

Mutual information (MI) is a measure of the amount of information that two random variables share. It quantifies the degree to which knowledge of one variable reduces uncertainty about the other variable. For communication signals, the higher the MI between the transmitter and the receiver is, the better the communication quality is. This means that more information is correctly transmitted. The Shannon limit is used to measure the channel capacity, by calculating the MI between the signals before entering the channel and the signals when leaving the channel. However, the receiver will still cause loss in MI. Therefore, the signals used in the calculation are expanded into bit sequences, as shown in Fig. 1, and the information rate is calculated based on GMI.

**Fig. 2:**
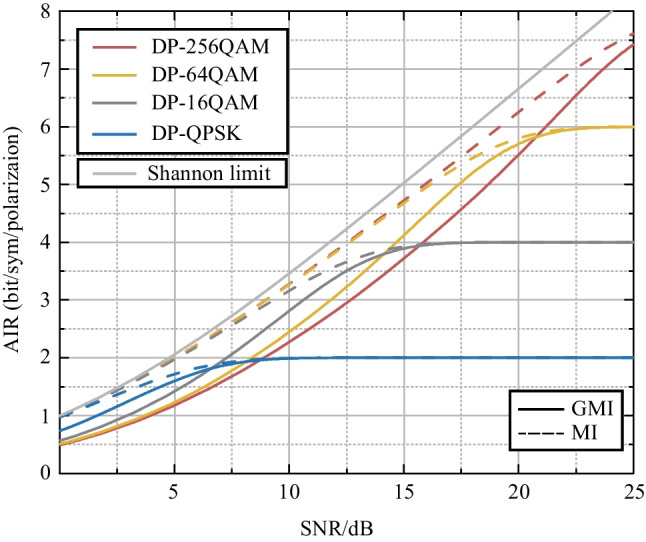
GMI and MI of DP-QPSK, DP-16QAM, DP-64QAM and DP-256QAM, *DP*: dual polarization

Suppose that the modulated bit signal at time *l* is $\{c_{1,l}, c_{2,l},..., c_{m,l} \}$. Every *m* bits will be mapped into one symbol $x_l$ and $x_i \in X, card(X)=M$, where *M* is order of the modulation format and *X* is modulated symbol set. The symbol obtained after passing through the channel is $y_i$, where $y_i\in Y$. Then the symbols will be demapped to bit sequences $\{L_{1,l}, L_{2, l},..., L_{m,l} \}$. MI and GMI can be estimated using following formulas [8]

1 $$\begin{aligned} MI&= I(X:Y) \nonumber \\&= \frac{1}{M} \sum ^M_{i=1} \int _{\mathcal {C}^N} f_{Y \vert X}(y\vert x_i)\log _2 \frac{f_{Y\vert X}(y\vert x_i)}{\frac{1}{M}\sum ^M_{j=1}f_{Y\vert X}(y\vert x_j)} \text{d}y, \end{aligned}$$

2 $$\begin{aligned} GMI&= \sum ^m_{k=1} \mathbb {E}_{B_k,Y}\left[ \log _2 \frac{f_{Y\vert B_k}(Y\vert B_k)}{\frac{1}{2}\sum _{b\in \{0,1\}}f_{Y\vert B_k}(Y\vert b)} \right] \nonumber \\&= \frac{1}{M} \sum ^m_{k=1} \sum _{b\in \{0,1 \}} \sum _{i\in \mathcal {I}_m^b} \nonumber \\&\int _{\mathcal {C}^N} f_{Y\vert X}(y\vert x_i)\log _2 \frac{\sum _{j\in \mathcal {I}_k^b} f_{Y\vert X}(y\vert x_j)}{\frac{1}{2}\sum _{p=1}^M f_{Y\vert X}(y\vert x_p)} \text{d}y, \end{aligned}$$

where $\mathcal {I}_m^b \subset \{1,2,..., M \}$ with $card(\mathcal {I}_m^b)=M/2$ is the set of indices of constellations points, of which the binary label is *b* at the bit position *m*. $f_{Y\vert X}(y\vert x)$ is the channel transfer function and $\mathcal {C}^N$ is the *N*-dimension constellation map. $B_{k}$ is the bit sequence. $\mathbb {E}$ is the expection. In practice, GMI and MI can be calculated using the Monte-Carlo integration method and the results are shown in Fig. 2 [9].

## Enhanced Gaussian noise model

Due to the existence of nonlinear effects, signal propagation in the fiber is very complicated. It is impossible to provide explicit expressions for signal transitions. However, the nonlinear effects of the channel are not very strong near the optimal power, where the behavior of signal propagation is close to linear signal propagation. This is the basic assumption of the perturbation-based Gaussian noise model. Poggiolini et al. proposed the EGN model for quickly estimating the SNR of optical fiber communication systems [10, 11]. In this paper, the EGN model is used to quickly calculate the channel SNR, and then the EGN-based evaluation of the corresponding nonlinear interference is added to estimate the system GMI. The EGN model in the C-band can be approximately expressed as [12, 13]

3 $$\begin{aligned} \text{SNR}&= \frac{P}{\sigma ^2+\sigma ^2_\text{s-s}+\sigma ^2_\text{s-n}}, \end{aligned}$$

4 $$\begin{aligned} \sigma ^2&=\sigma _\text{TRx}^2+\sigma _\text{ASE}^2, \end{aligned}$$

5 $$\begin{aligned} \sigma ^2_\text{s-s}&= N_\text{s}^{\epsilon +1}\eta P^3, \end{aligned}$$

6 $$\begin{aligned} \sigma ^2_\text{s-n}&\approx 3\left(\frac{N_\text{s}^{\epsilon +1}}{2}+\frac{N_\text{s}^{\epsilon +2}}{\epsilon +2}\right)\eta \sigma ^2_\text{ASE} P^2 + 3N_\text{s}^{\epsilon +1}\eta \kappa P^3, \end{aligned}$$

where *P*, $\sigma ^2_\text{ASE}$, $\sigma ^2_\text{TRx}$ are the signal power, the amplifier spontaneous emission (ASE) noise power and the transceiver noise power, respectively. $N_\text{s}$ is the number of fiber spans.

7 $$\begin{aligned} \epsilon&= \frac{3}{10}\log \left[ 1+\frac{6}{L_{\text{s}}}\frac{L_{\text{eff}}}{\sinh ^{-1}(\frac{{{\uppi}} }{2}\vert \beta _{2}\vert R^2_{\text{s}}N^2_\text{ch}L_{\text{eff})}}\right], \end{aligned}$$

8 $$\begin{aligned} \eta&\approx \frac{8}{27}\frac{\alpha \gamma ^2L_{\mathrm{{eff}}}}{{{\uppi}} \vert \beta _{2}\vert R_{\mathrm{{s}}}^2} \sinh ^{-1} \left( \frac{{{\uppi}} ^2}{2}\vert \beta _{2}\vert L_{\mathrm{{eff}}}N_{\mathrm{{ch}}}^2R_{\text{s}}^2\right) \\&-\frac{80}{81}\frac{\kappa \gamma ^2L_{\mathrm{{eff}}}^2}{{{\uppi}} \vert \beta _{2}\vert L_{\mathrm{{s}}}R_{\mathrm{{s}}}^2}\left[ \Phi \left( \frac{N_{\mathrm{{ch}}}+1}{2}\right) +C+1\right], \end{aligned}$$

where $L_\text{eff}=(1-\text{e}^{-\alpha L_\text{s}})/\alpha$, $\alpha$ is the fiber attenuation coefficient, $\beta _2$ is the second-order dispersion coefficient, $N_\text{ch}$ is the number of WDM channels, $R_\text{s}$ is the symbol rate, $C\approx 0.557$ is the Euler-Mascheroni constant and $\gamma$ is fiber nonlinear coefficient. $L_\text{s}$ is the span length. $\Phi (x)$ is the digamma function and $\kappa$ is a constant equals to 1, 17/25, 13/21, and 121/200 corresponding to QPSK, 16QAM, 64QAM, and 256QAM, respectively [11]. The accuracy of EGN model in the C-band has already been verified by other scholars as well in our previous works [14–17].

## Results and discussion

**Fig. 3:**
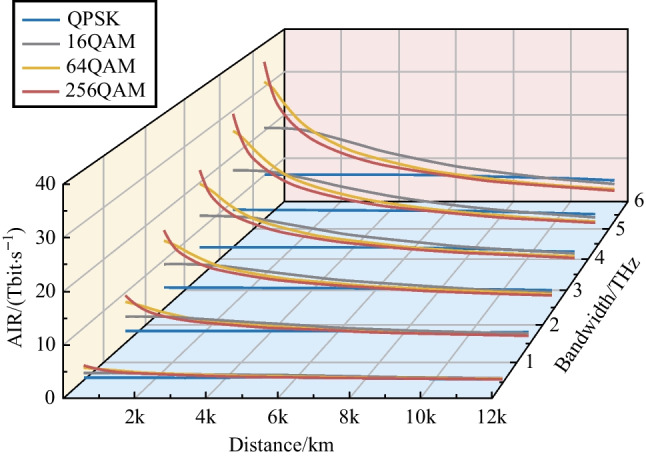
AIR versus transmission distance and communication bandwidth. Symbol rate is 32 GBaud and each fiber span is 80 km

For a Nyquist-spaced optical communication system, according to the Nyquist sampling theorem, the number of symbols transmitted per second can be measured via the bandwidth of the system. The value of GMI represents the effective number of bits in a symbol. Multiplying the bandwidth by the GMI gives the effective number of bits per second, transmitted over each polarization mode. This paper studies the communication scenario of a 80 km per span 32 GBaud fiber communication system with different modulation formats, transmission distances and bandwidths. Results of AIR versus transmission distances and bandwidths are shown in Fig. 3.

**Fig. 4:**
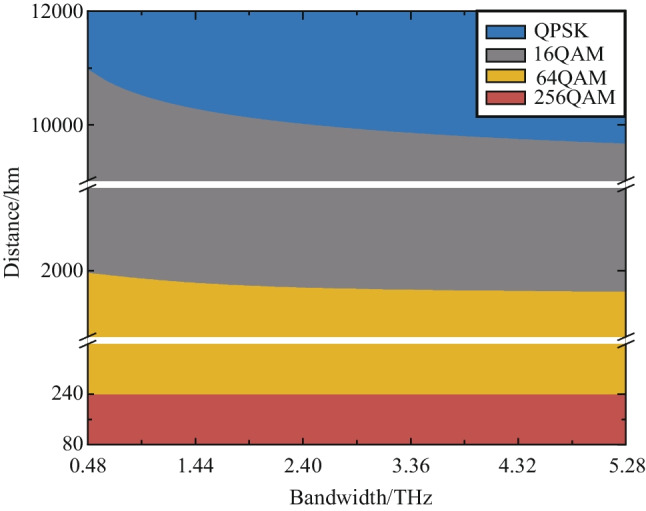
The optimal modulation formats under different transmission distances and communication bandwidths. Symbol rate is 32 GBaud and each fiber span is 80 km

The MI degradation at the receiver is particularly severe for higher-order modulation formats, as shown in Fig. 2. When the SNR is low, the GMI of the high-order modulation format drops sharply, and it can be even lower than that of the low-order format at low SNR region. Moreover, higher-order modulation formats are more significantly affected by the noise, resulting in a more severe GMI degradation. It is demonstrated that higher-order modulation formats show their advantages in the case of shorter transmission distances or smaller communication bandwidths. For systems with long transmission distances and large bandwidths, some low-order modulation formats can be more robust and appropriate. Figure 4 shows the optimal modulation format for different transmission situations.

For terrestrial communication systems, the common fiber span length is 80 km, and the transmission distance is less than 10000 km. When the symbol rate is 32 GBaud and the transmission distance exceeds 2000 km, the modulation format of 16QAM can always obtain the highest AIR. When the transmission distance is reduced to between 240 and 2000 km, the modulation scheme of 64QAM becomes the most suitable format. The 256QAM signal can surpass the other three modulation formats only when the transmission distance is lower than 240 km.

**Fig. 5:**
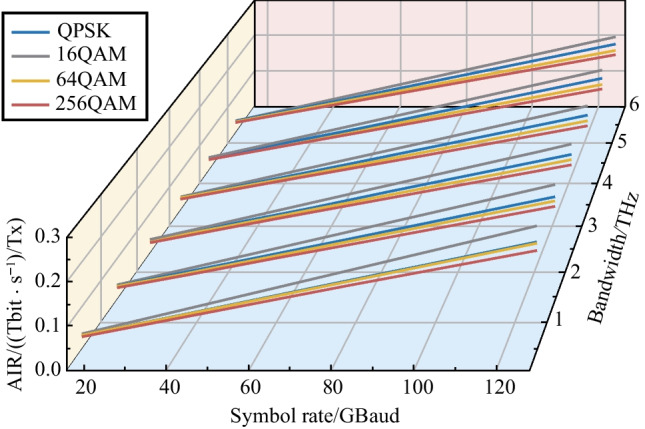
AIR per transmitter versus symbol rate and communication bandwidth. Transmission distance is 8000 km and each fiber span is 80 km

To study higher symbol rate systems, we fixed the transmission distance as 8000 km. Figure 5 shows the GMI with different symbol rates and different communication bandwidths at transmission distance of 8000 km and fiber span of 80 km.

Every curve in Fig. 5 is almost as straight line, and this means that the GMI is weakly correlated with the symbol rate. However, increase of the communication rate can save the number of channels for WDM transmission and hence saves the related component sets cost. Therefore, higher-speed transmitters have more efficient AIR per transmitter. Meanwhile, the GMI behaves almost independently of the symbol rate, and so 16QAM can still obtain the best performance at 8000 km as shown in Fig. 4.

**Fig. 6:**
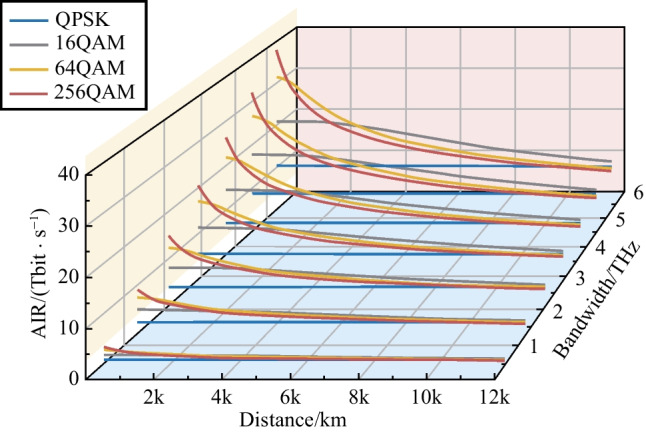
AIRs versus transmission distances and communication bandwidths. Symbol rate is 32 GBaud and each fiber span is 50 km

A submarine communication system with a span length of 50 km is also studied. Compared with the system with 80 km span, shortening the span to 50 km can significantly improve the system SNR [14], so the higher-order modulation formats could benefit from this. The result is shown in Fig. 6.

The intersection between curves with different colors in the same group moves towards a longer communication distance when higher-order modulation is used. This proves that the higher-order modulation format obtains more improvement than the lower-order format with the increase of system SNR. Since current transmission scenario refers to a submarine system, we focus on the scenario where the communication distance exceeds 8000 km. When the span length is 50 km, it can be found that the QPSK modulation format can almost reach the maximum GMI (2 bit/sym/polarization). This is also the reason why QPSK format is widely used in current submarine communications. However, the 16QAM modulation format also obtains a great improvement, and the use of the 16QAM format within 12000 km can significantly improve the system AIR, especially for larger bandwidth.

In summary, symbol rate has little effect on the system GMI, but the application of a higher symbol rate can effectively reduce the number of required transceivers and link components. For long distance (2000–10000 km) terrestrial communication systems with 80 km per span, the 16QAM format can get the highest AIR. For the submarine communication systems with each fiber span of 50 km [18], the 16QAM shows more significant performance improvement compared to the QPSK format. In a terrestrial communication system or a submarine communication system, it can be seen that the communication bandwidth has marginal effects on the SNR, as shown in Fig. 5. Therefore, trade-off between high-speed transmitters and the number of channels is important when designing new fiber optic systems. For the convenience of use, we list the results (optimal selection of the modulation format) for bandwidth exceeding 2.4 THz as in the following Tables 1 and 2.

**Table 1:** Optimal modulation format for bandwidth of over 2.4 THz and span distance of 80 km

Transmission distance	Modulation format
< 240 km	256 QAM
240–2000 km	64 QAM
2000–10000 km	16 QAM
> 10000 km	QPSK

**Table 2:** Optimal modulation format for bandwidth of over 2.4 THz and span distance of 50 km

Transmission distance	Modulation format
< 500 km	256 QAM
500–3000 km	64 QAM
3000–12000 km	16 QAM
> 12000 km	QPSK

## Proposals for the future

The MI of the high-order modulation format is always higher than that of the low-order format. However, the GMI of the higher-order modulation format could be lower than that of the lower-order format due to the loss of information caused by the transceiver. Therefore, the use of more advanced transceivers can be an effective solution. In fact, the SNR difference between each modulation format is very small, especially when the modulation order is higher than 4 (equal to or above 16QAM) [19]. Various methods that can reduce the information loss at the receiver side or shift the intersection between solid lines with different colors (modulation formats) to the left (low SNR region) in Fig. 2 will be an interesting research direction for next-generation fiber communication systems. On the other hand, another hot research direction use various approaches, such as constellation shaping and waveform shaping [20], to improve the GMI of the optical fiber system, thereby shifting the dotted line in Fig. 2 closer to the Shannon limit (the gray line). Optical fiber communication systems, despite still having a long way to go, will eventually become the cornerstone of future telecommunication networks.

## Data Availability

The data that support the findings of this study are available from the corresponding author, upon reasonable request.
